# ChoroidSeg-ViT: A Transformer Model for Choroid Layer Segmentation Based on a Mixed Attention Feature Enhancement Mechanism

**DOI:** 10.1167/tvst.13.9.7

**Published:** 2024-09-05

**Authors:** Zhaolin Lu, Tao Liu, Yewen Ni, Haiyang Liu, Lina Guan

**Affiliations:** 1 The Affiliated Xuzhou Municipal Hospital of Xuzhou Medical University, Xuzhou, Jiangsu, China; 2 School of Information and Control Engineering, China University of Mining and Technology, Xuzhou, Jiangsu, China; 3 Xuzhou Medical University, Xuzhou, Jiangsu, China

**Keywords:** choroid layer segmentation, deep learning, transformer

## Abstract

**Purpose:**

To develop a Vision Transformer (ViT) model based on the mixed attention feature enhancement mechanism, ChoroidSeg-ViT, for choroid layer segmentation in optical coherence tomography (OCT) images.

**Methods:**

This study included a dataset of 100 OCT B-scans images. Ground truths were carefully labeled by experienced ophthalmologists. An end-to-end local-enhanced Transformer model, ChoroidSeg-ViT, was designed to segment the choroid layer by integrating the local enhanced feature extraction and semantic feature fusion paths. Standard segmentation metrics were selected to evaluate ChoroidSeg-ViT.

**Results:**

Experimental results demonstrate that ChoroidSeg-ViT exhibited superior segmentation performance (mDice: 98.31, mIoU: 96.62, mAcc: 98.29) compared to other deep learning approaches, thus indicating the effectiveness and superiority of this proposed model for the choroid layer segmentation task. Furthermore, ablation and generalization experiments validated the reasonableness of the module design.

**Conclusions:**

We developed a novel Transformer model to precisely and automatically segment the choroid layer and achieved the state-of-the-art performance.

**Translational Relevance:**

ChoroidSeg-ViT could segment precise and smooth choroid layers and form the basis of an automatic choroid analysis system that would facilitate future choroidal research in ophthalmology.

## Introduction

The choroid is a vascular structure situated between the retina and the sclera. It plays a vital role in the human visual system and provides adequate nutrition to the outer retina.^1^ In recent years, ophthalmologists have observed that the onset of and deterioration associated with ophthalmic diseases are closely related to choroidal structure changes. Choroidal thickness and vascularity have emerged as biomarkers of various ocular diseases, such as glaucoma,^2^ diabetic retinopathy (DR),^3^ and age-related macular degeneration (AMD).^4^ For example, studies have revealed that choroid layer thickening and retinal layer thinning significantly correlate with the choroidal vascularity index. Additionally, a negative correlation exists between the severity of diabetic retinopathy and the choroidal vascularity index. Therefore, precise segmentation of the choroid layer is essential for comprehensive analysis and understanding of choroidal changes.

Medical imaging is currently the primary method for clinical diagnosis and determining the prognosis and treatment of diseases, among which optical coherence tomography (OCT) is widely applied to ophthalmic disease diagnosis due to its being non-invasive and convenient.^5^ The utilization of OCT imaging to observe choroidal structural features is crucial for accurate diagnosis and timely treatment. Accurate segmentation of the choroid layer is a vital component of analyzing the choroidal structure. Nevertheless, it is a challenging task due to the difficulty of distinguishing the fuzzy choroid–scleral interface (CSI) from the choroid.^6^ Traditional medical examinations typically involve time-consuming and error-prone manual labeling methods, which are unsuitable for meeting the growing clinical needs.

The field of medical lesion segmentation is seeing an increasing utilization of image segmentation algorithms based on image processing and deep-learning technologies, with a particular focus on choroid layer segmentation. This can be achieved primarily through either graph-based segmentation methods or learning-based segmentation methods.^7^^–^^9^ Graph-based methods have been extended from graph search algorithms by applying graph cuts,^10^ max-flow min-cut graph theory,^11^ and level set^12^ to detect choroid boundaries in OCT images. These methods heavily rely on handcrafted features and complex segmentation pipelines, resulting in limited segmentation accuracy and robustness.

With the complexity of segmentation scenes and the difficulty of feature learning increasing, convolutional neural networks (CNNs) have been widely utilized in medical image analysis due to their ability to automatically extract image features.^13^^–^^16^ To accurately segment choroidal boundaries, Sui et al.^17^ proposed an end-to-end convolutional architecture that learns the optimal graph boundary weights directly from raw OCT images. Masood et al.^18^ combined convolutional networks and morphological studies to establish a patch-based architecture, but the overly complex training process limits its segmentation efficiency. Recently, Khaing et al.^19^ developed a dense dilated U-Net model (ChoroidNET) by integrating dilated convolution with varying rates into the U-Net model to precisely segment choroid layers and choroidal vessels in spectral-domain OCT (SD-OCT) images. Wu et al.^20^ proposed a framework for choroid segmentation that enhances boundary features by incorporating expert knowledge and soft point maps of features. This approach achieves reasonable segmentation of the choroid layer on both two-dimensional (2D) and three-dimensional (3D) OCT images.

In recent years, Vision Transformers (ViTs) have achieved remarkable success in various computer vision tasks, including image classification, object detection, and semantic segmentation. This success can be attributed to their excellent ability of modeling global relationships.^21^^,^^22^ Chen et al.^23^ embedded Transformer blocks^24^ into the U-Net encoder path to capture global contextual information; however, their performance relies heavily on numerous medical images and significant computational costs. To efficiently train Transformer models on medical images, Valanarasu et al.^25^ proposed a local–global training strategy that processes entire images and patches separately to capture global and local features. Although the aforementioned Transformer models exhibit global feature extraction capabilities, they simultaneously suppress the learning of detailed features. Therefore, the integration of CNNs and Transformers in a hybrid structure has emerged as a novel paradigm for complex segmentation tasks, effectively combining global and local features to facilitate the segmentation performance. HRFormer^26^ integrates Transformer blocks into the HRNet,^27^ allowing the model to benefit from both the multi-resolution feature fusion architecture and the ability to capture global semantic information via modeling long-range dependencies. CMT^28^ combines the MobileNet architecture with Transformer blocks in a parallel manner, enabling bidirectional fusion of local and global features. This novel approach achieves high performance in downstream tasks, surpassing that of lightweight networks such as MobileNetV3.^29^

In this study, we focus on tackling two issues in choroid layer segmentation. Firstly, existing methods typically expand the receptive field blindly to obtain global contextual information, which inhibits them from learning local details and leads to inaccurate boundary segmentation. Secondly, there is a lack of efficient utilizing multi-scale feature information, resulting in existing methods unable to fully learn the detailed features that distinguish fuzzy boundaries. To solve the issues mentioned above, this study combines advantages of inductive bias and efficient receptive field of both CNN and ViT, and proposes a choroid layer segmentation model based on the mixed attention feature enhancement mechanism. This model utilizes multi-scale information to learn the feature distinctions between different layer structures via introducing the local-enhanced feature extraction and semantic feature fusion paths, thereby enhancing the learning and representation ability of choroid boundary features.

## Methods

### Datasets and Implementation Details

This study utilized two datasets to conduct subsequent experiments: the Choroid-DS and the public Retinal OCT-C8.^30^ The data in the Choroid-DS were obtained from choroid images captured by the DRI OCT-1 system (Topcon, Tokyo, Japan) at the First People's Hospital of Xuzhou City. This dataset encompassed a total of 100 2D enhanced depth imaging OCT B-scan images derived from 75 patients, including 41 males and 34 females. Forty-seven patients were over 60 years of age, and 28 patients were under 60 years of age. Notably, the 100 OCT images were classified into two categories: DR and normal. Specifically, 68 images belonged to the normal OCT category, and 32 images were classified as DR images. The images in the dataset were annotated by experienced ophthalmologists using the LabelMe annotation software. For each class in the dataset, the data were divided into training, validation, and testing subdatasets in an 8:1:1 rate to ensure that each subdataset contained both DR and normal images.

The Retinal OCT-C8 dataset contains 24,000 2D OCT B-scan images in eight categories with three different image resolutions. In this study, the 1000 images selected from the dataset were utilized to evaluate the cross-dataset generalization ability of our model. These included 200 images each of AMD, DR, diabetic macular edema (DME), drusen, and normal. Given the absence of label images in the dataset, we proceeded to annotate the 1000 images at the pixel level. The images were then divided into training, testing, and validation subdatasets at an 8:1:1 rate.

In the experiments, each image and its corresponding label map in the datasets were reshaped to a resolution of 512 × 512 in order to reduce computational complexity without compromising choroidal visibility. Data enhancement was performed on the raw datasets using horizontal flipping, angular transformation, and contrast enhancement to reduce overfitting problems and to improve the generalization ability of the model. The proposed model was evaluated every 2000 iterations during the 80,000 experimental training iterations. The training hyper-parameters for the model was set as follows: Adam optimization, with an initial learning rate of 0.0005 and a batch size of 4, and the binary cross-entropy loss, which was selected as the loss function. The training strategies employed for other comparison methods were consistent with those outlined in their original studies. For the fairness of the experimental results, the same auxiliary segmentation head as that of the Transformer network was added on top of the original convolutional networks. The deep-learning methods used in the experiments were implemented via the PyTorch MMSegmentation model library and were run on a single Quadro RTX 6000 GPU (NVIDIA, Santa Clara, CA). To reduce bias and increase statistical significance, the segmentation results were consistently cross-validated fivefold across all methods.

To compare the performance of the proposed model with other state-of-the-art deep- learning methods, three key evaluation metrics were used to measure the degree of similarity between the predicted masks and the ground truths: Intersection over Union (IoU), Dice coefficient (Dice), and accuracy (Acc).

(1) $$\begin{array}{c} IoU=\frac{\mathrm{TP}}{\mathrm{TP}+\mathrm{FP}+\mathrm{FN}} \end{array}$$

(2) $$\begin{array}{c} Dice=\frac{2\times \mathrm{TP}}{2\times \mathrm{TP}+\mathrm{FP}+\mathrm{FN}} \end{array}$$

(3) $$\begin{array}{c} Acc=\frac{\mathrm{TP}+\mathrm{TN}}{\mathrm{TP}+\mathrm{TN}+\mathrm{FP}+\mathrm{FN}} \end{array}$$

where the variables *TP*, *FP*, *TN*, and *FN* represent true positive, false positive, true negative, and false negative, respectively. In subsequent experiments, mIoU, mDice, and mAcc were utilized as the evaluation metrics, where the prefix “m” denotes the average value of the choroid layer and background segmentation metrics.

### ChoroidSeg-ViT

**Figure 1.**
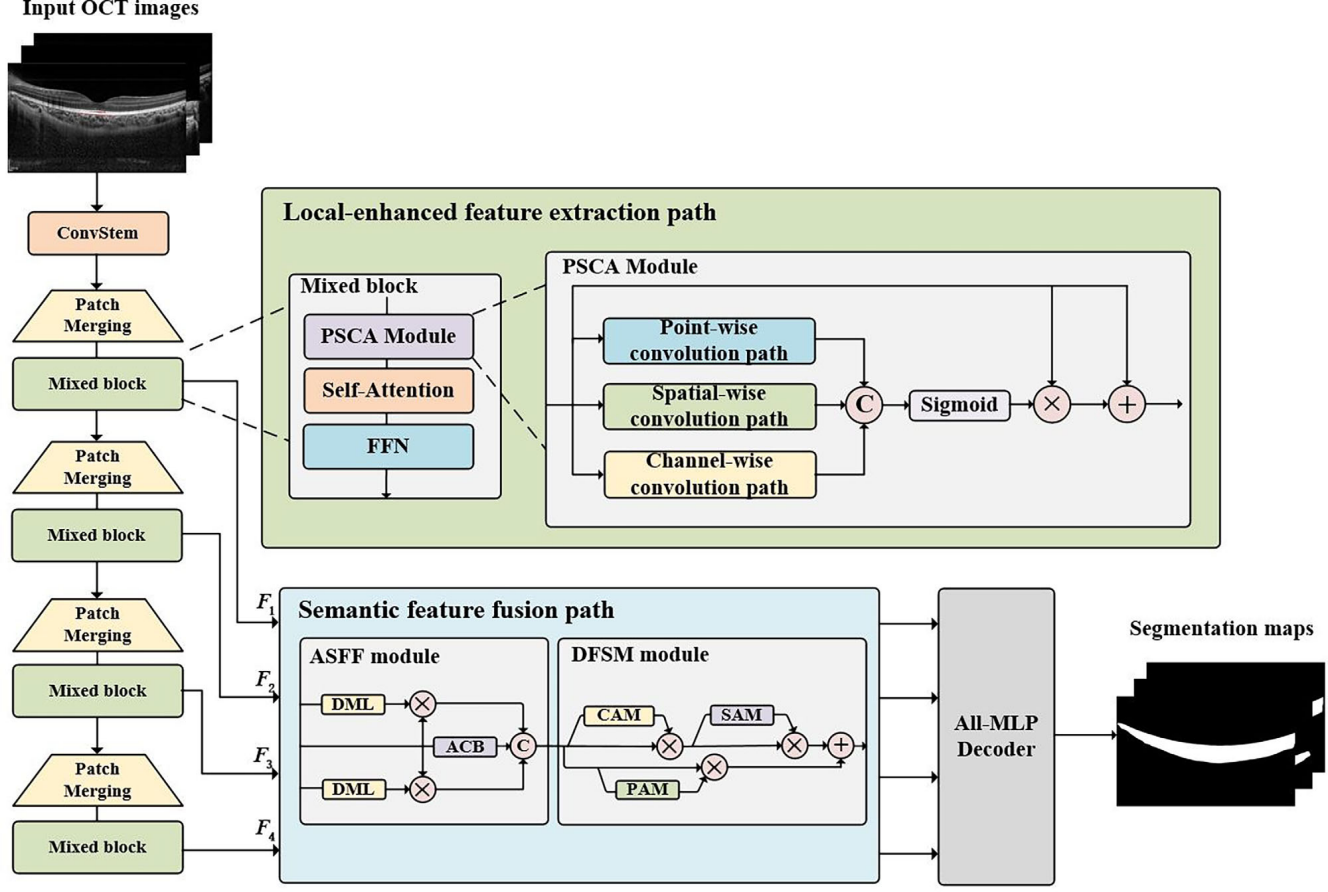
Network architecture of the ChoroidSeg-ViT.

The ChoroidSeg-ViT is comprised of three main components: a local-enhanced encoder, a semantic feature fusion path, and a segmentation decoder, as shown in Figure 1. The encoder follows a hierarchical structure and utilizes mixed blocks that integrate the parallel multiscale convolutional attention (PSCA) module and self-attention module to construct a local-enhanced feature extraction path. This path can enhance the capacity of capturing local information on the basis of global representation. Downsampling operations are performed by Patch Merging layers, gradually extracting low-level detail features and high-level semantic features. The semantic feature fusion path consists of the adjacent multiscale feature fusion (ASFF) module and the dynamic feature selection module (DFSM), in which the ASFF module efficiently fuses adjacent hierarchical multiscale features to facilitate the information interaction between high-level semantic features and low-level detailed features, and the DFSM dynamically selects key features with semantic consistency from the fused features while suppressing redundant ones. The decoder path adopts an all-multilayer perceptron (MLP) decoder to improve the segmentation performance with lower computational complexity. The last layer of the decoder employs a linear layer to classify foreground and background pixels, resulting in the segmentation maps for choroid layer segmentation.

#### Local Enhanced Encoder

The ChoroidSeg-ViT adopts a local enhanced hierarchical encoder consisting of four feature extraction stages with varying spatial resolutions. Each stage includes a stack of a downsampling layer (Patch Merging layer) and a series of local enhanced feature extraction blocks (Mixed block). Specifically, a downsampling layer consists of a 3 × 3 depth-wise convolution (DWConv) layer and a batch normalization (BN) layer, which gradually expands receptive fields to capture deep semantic features. The feature extraction block employs the local enhanced feature extraction path, comprised of a PSCA, a multi-head self-attention (MHSA) module, and a feedforward network (FFN) module. The PSCA is used to enhance the ability of local information extraction on the basis of global information modeling of the MHSA. The FFN is employed to enhance the nonlinearity of self-attention computations and boost the representational capacity of the model. After multiple downsampling and feature extraction layers, the hierarchical encoder outputs multiscale feature series with different resolutions, which can be denoted as [*F*_1_, *F*_2_, *F*_3_, *F*_4_].

##### Parallel Multiscale Convolutional Attention Module

**Figure 2.**
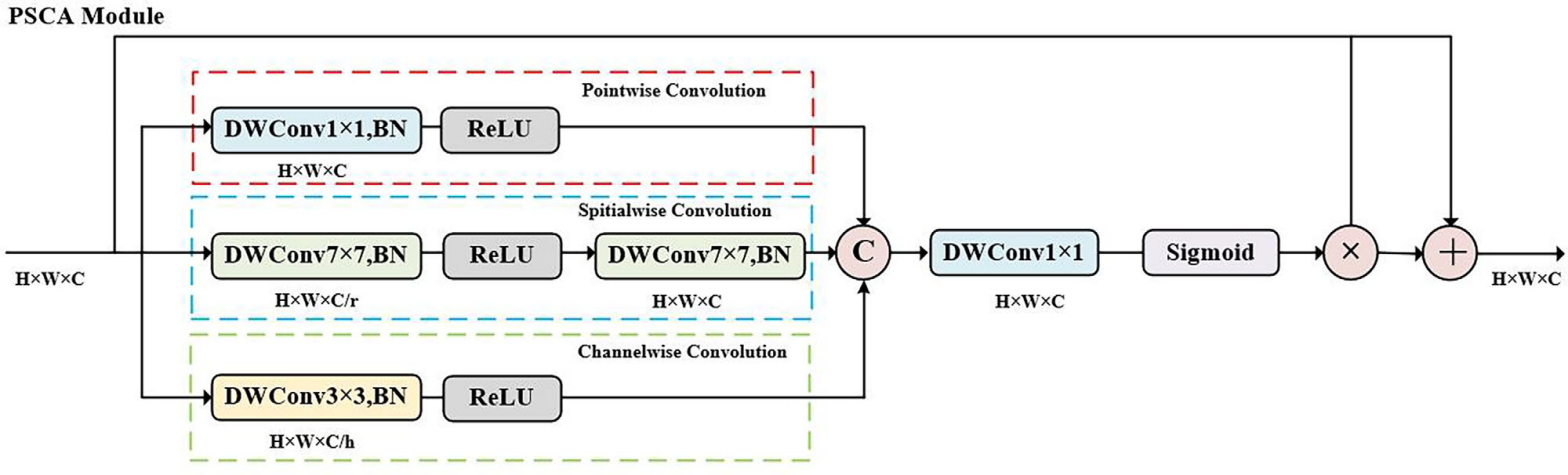
Detailed structure of the PSCA module.

Recent studies have attempted to introduce convolutional algorithms into ViT architectures to improve their segmentation performance. However, the introduction of a simple convolutional layer or module may not be enough to capture detailed features required for complex medical segmentation tasks. As shown in Figure 2, the PSCA module is designed by combining multiple convolution operations to construct three convolutional paths with different receptive fields in parallel to extract abstract features: a pointwise convolution path (1 × 1 DWConv), a channel-wise convolution path (3 × 3 DWConv), and a spatial-wise convolution path (two 7 × 7 DWConvs in series). First, three convolution paths capture multiscale features with varying receptive fields and dimensions. The output feature maps of each path are then fused using a concatenation operation. After feature fusion, the number of channels is compressed using a 1 × 1 convolution layer to ensure consistency with the original input channels. The attention weights for each dimension are calculated using a sigmoid activation function. These weights are subsequently multiplied with original features to obtain the attention-weighted features. To avoid the loss of important information caused by convolution and channel compression operations, a residual connection is utilized to sum the weighted features with the input features, resulting in the final output features. To reduce the computational complexity, the PSCA module adopts a lightweight design that uses depth-separable convolutions to construct the parallel convolution paths, and the group number of depth-separable convolutions is set to be the same as the channel numbers. Also, a compression ratio of 16 is used in the spatial-wise convolution path to compress channel dimensions.

##### Semantic Feature Fusion Path

In the task of choroid layer segmentation, reducing semantic differences of features at varying scales by fusing coarse-grained features and fine-grained features is beneficial to improve the segmentation accuracy of the model. To this end, a semantic feature fusion path that consists of the ASFF module and the DFSM is designed to effectively fuse multiscale features with varying semantic information and dynamically select prominent features with high semantic consistency.

##### Adjacent Multiscale Feature Fusion Module

**Figure 3.**
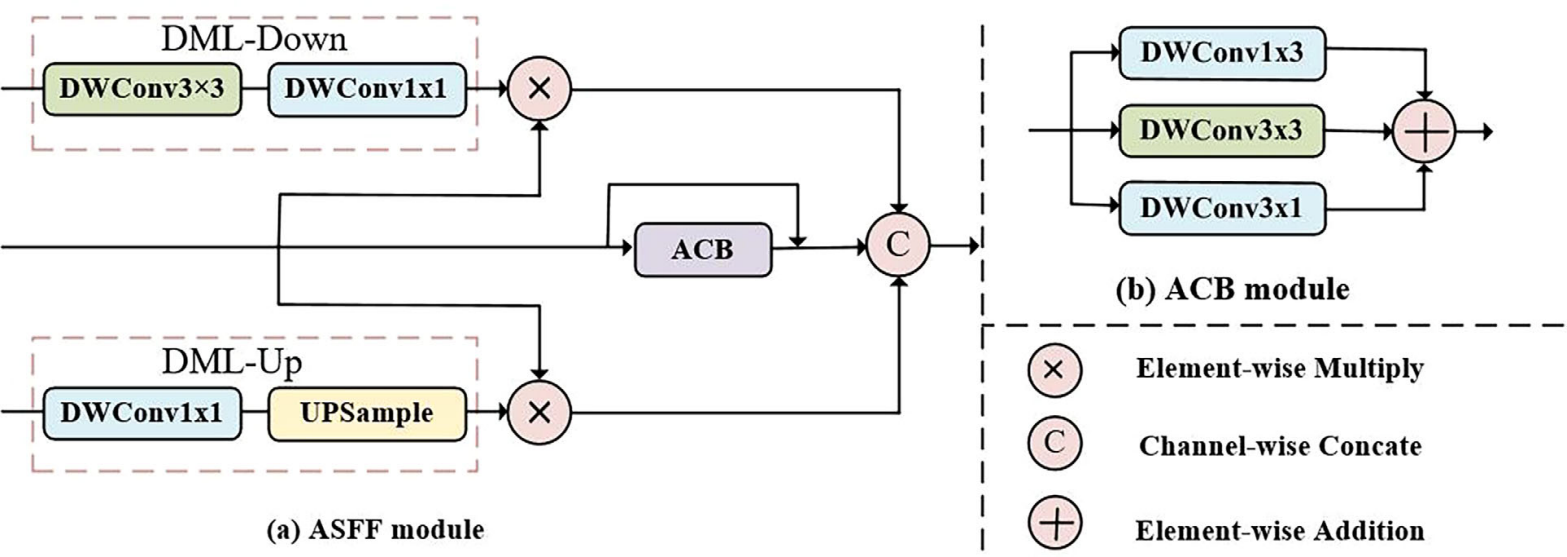
Detailed structure of the ASFF module. (**a**) illustrates the workflow of the ASFF module, while (**b**) depicts the the ACB module that integrated into the ASFF module.

The ASFF module is designed based on a simple and effective structure, which guides the feature selection of the current layer through the low-level detail features and high-level semantic features provided by the adjacent layers. The design of the ASFF is such that the feature map sizes of adjacent layers are unified via a simple dimension matching layer (DML), and the multiscale feature fusion between different layers is realized by using the concatenate operation. In addition, an asymmetry convolution block (ACB)^31^ is embedded in a residual connection to deeply mine the semantic information. As shown in Figure 3b, the ACB module splits a 3 × 3 convolution into 3 × 3, 1 × 3, and 3 × 1 convolutions and then concatenates the feature maps from three convolutions to obtain the final result. Among them, the 1 × 3 and 3 × 1 convolutions share parameters with the 3 × 3 convolution to strengthen key features with zero parameter increase.

In the structure of ChoroidSeg-ViT, the ASFF module is utilized to fuse the multiscale features output from the encoder [*F*_1_, *F*_2_, *F*_3_, *F*_4_]. For *F*_1_ and *F*_4_, the ASFF module is applied to fuse adjacent layer features; for the intermediate feature layers [*F*_2_, *F*_3_], the ASFF module is used to integrate the multiscale features from three adjacent stages. Taking the intermediate layer encoder output *F_i_* (*i* = 2, 3) as an example, the ASFF module first performs dimension matching operations on the low-resolution features *F_i_*_+1_ and high-resolution features *F_i_*_–1_. For low-resolution features *F_i_*_+1_, a 1 × 1 convolution layer is used to compress the number of channels by halves, and then an upsample layer (bilinear interpolation) is applied to expand the feature map size to the same as *F_i_*, resulting in the transformed feature $F_{i+1}^{T}$. For high-resolution features *F_i_*_–1_, a 3 × 3 depth convolution layer and a 1 × 1 layer are adopted to reduce the spatial resolution by half and match the channel number, separately. For the current feature *F_i_*, the enhanced feature $F_{i}^{T}\;$is obtained by using an ACB module and a residual connection to deeply mine multiscale semantic information and retain more detailed features. After dimension matching, element-wise multiplications of $F_{i-1}^{T}\;$and $F_{i+1}^{T}\;$with $F_{i}^{T}\;$are conducted separately to enhance distinctive features and suppress background noise. Finally, a concatenate operation is performed to fuse the multiscale feature output from adjacent layers, capturing the correlation information among the multiscaled features to obtain the final fused features. The formulae are expressed as follows:

(4) $$\begin{array}{c} F_{i}^{T}=F_{i}+ACB\left(F_{i}\right),\;\left(i=1,2,3,4\right) \end{array}$$

(5) $$\begin{array}{c} F_{i+1}^{T}=UP\left(Conv_{1\times 1}\left(F_{i+1}\right)\right),\;\left(i=2,3,4\right)\; \end{array}$$

(6) $$\begin{array}{c} F_{i-1}^{T}=Conv_{1\times 1}\left(DWConv\left(F_{i-1}\right)\right),\;\left(i=1,2,3\right)\; \end{array}$$

(7) $$\begin{array}{ccc} F_{i}^{ASFF} & = & Concat(Mul\left(F_{i-1}^{T},F_{i}\right),F_{i}^{T},\\ & & Mul\left(F_{i},F_{i+1}^{T}\right)),\;\left(i=1,2,3,4\right)\; \end{array}$$

where *UP* denotes a twofold up-sample operation; *DWConv* represents a 3 × 3 depthwise convolution and denotes an asymmetric convolution module; and *Mul* and *Concat* represent a pixel-by-pixel multiplication operation and a concatenate operation, respectively.

##### Dynamic Feature Selection Module

Inspired by the convolutional block attention module (CBAM),^32^ which combines spatial and channel attention, this study designed a DFSM that integrates three different attention mechanisms (namely, spatial attention, channel attention, and point attention), thus enabling the model to dynamically select features that carry similar semantic information. The DFSM weights the feature map output from ASFF in three different dimensions to adequately measure the importance of each feature, dynamically selects highly weighted features, and reduces feature redundancy. Unlike the CBAM, the DFSM further calculates point attention at each feature point on the basis of redesigning the spatial attention module and channel attention module, so as to improve the segmentation performance for fuzzy boundary regions of the choroid with less computational complexity.

**Figure 4.**
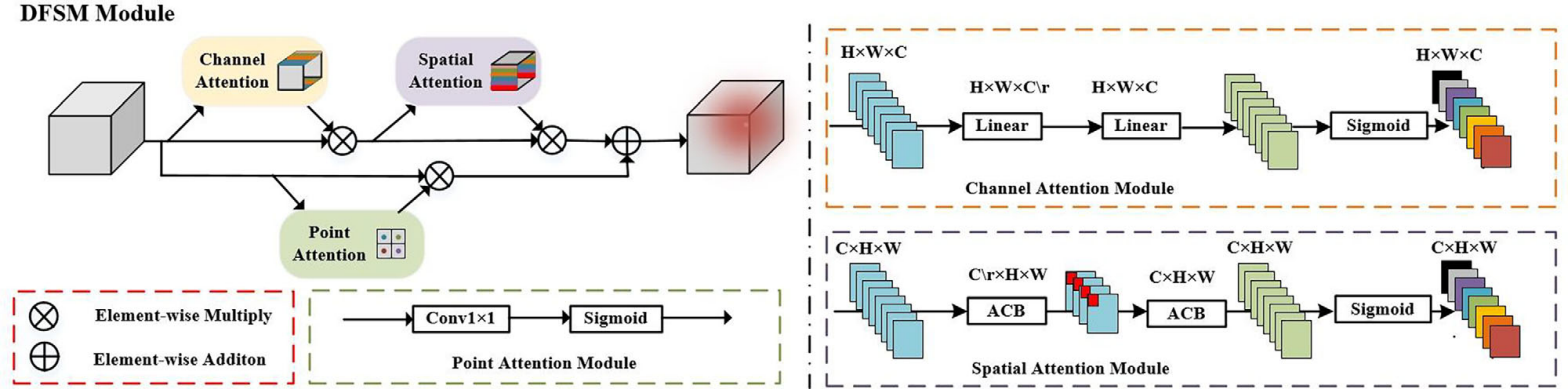
Detailed structure of the DFSM.

Formally, the DFSM combines three attention mechanisms to dynamically select important features with semantic consistency. Its specific structure is shown in Figure 4. First, the formula below can be used to calculate the channel-wise attention-weighted features:

(8) $$\begin{array}{c} A^{C}\left(F\right)=Sig\left(MLP\left(F,r\right)\right)\; \end{array}$$

where *A^C^*(*F*) denotes the output features of the channel attention submodule, *Sig* represents the Sigmoid activation function, *MLP* denotes the multilayer perceptron consisting of two linear layers, and *r* indicates the compression ratio of channel number. Next, the spatial-wise attention-weighted computational expression is

(9) $$\begin{array}{c} A^{S}\left(F\right)=Sig\left(ACB\left(ACB\left(F,r\right)\right)\right)\; \end{array}$$

where *A^S^*(*F*) denotes the output features of the spatial attention submodule, and *ACB* refers to an asymmetric convolution module consisting of three convolution kernels: 7 × 7, 7 × 1, and 1 × 7. Spatial feature fusion is performed on the features weighted by the channel attention using two ACB convolutional layers with large receptive fields. Finally, the pointwise attention weights are calculated by the following formula:

(10) $$\begin{array}{c} A^{P}\left(F\right)=Sig\left(Conv_{1\times 1}\left(F\right)\right)\; \end{array}$$

where *A^P^*(*F*) represents the output features of the point attention submodule. It weighs the importance of each feature point to perform feature selection in a point-to-point manner, thus improving the compatibility of multiscale features. After obtaining the attention weights for the three dimensions, the DFSM obtains the final attention-weighted features by using the following formulae:

(11) $$\begin{array}{c} F_{i}^{C}=A^{C}\left(F_{i}\right)⊗F_{i},\;i=\left(1,2,3,4\right)\; \end{array}$$

(12) $$\begin{array}{c} F_{i}^{S}=A^{S}\left(F_{i}^{C}\right)⊗F_{i}^{C},\;i=\left(1,2,3,4\right)\; \end{array}$$

(13) $$\begin{array}{c} F_{i}^{P}=A^{P}\left(F_{i}\right)⊗F_{i},\;i=\left(1,2,3,4\right)\; \end{array}$$

(14) $$\begin{array}{c} F_{i}^{DFSM}=Conv_{1\times 1}\left(F_{i}^{S}⊕F_{i}^{P}\right),\;i=\left(1,2,3,4\right)\; \end{array}$$

where $F_{i}^{C}$, $F_{i}^{S}$, and $F_{i}^{P}\;$separately denote the weighted features of the three attention submodules in the DFSM; *F_i_* indicates the output features from the ASFF module; and ⊗ and ⊕ represent the pixel-by-pixel multiplication and addition, respectively. In concrete terms, the output features from the ASFF module are passed through three attention submodules to obtain the attention weights in different dimensions and then multiplied with the original input to obtain the corresponding attention-weighted features. Finally, an addition operation is applied to fuse the weighted features from the channel–spatial attention path and point attention path, then a 1 × 1 convolution layer is used to restore the channel number to the same as the input, resulting in the final weighted features of the DFSM. The module is capable of dynamically selecting key features to recognize the choroid layer and reduces semantic ambiguities among multiscale features through weight reassignment.

## Results

### Comparison Experiments

This study conducted training, validation, and testing using the Choroid-DS dataset and compared the segmentation performance of the proposed ChoroidSeg-ViT with other mainstream deep-learning methods from both qualitative and quantitative perspectives. The comparison methods mainly include two categories: (1) CNN methods based on local representations (U-Net,^14^ DeepLabV3+,^33^ and PSPNet^34^), and (2) ViT methods based on global self-attention mechanisms (SegFormer,^35^ Next-ViT,^36^ and Hi-Former).^37^

#### Quantitative Results

To quantitatively evaluate the advantages of ChoroidSeg-ViT, Dice, IoU, and ACC were selected as evaluation metrics in this study. Table 1 presents the performance comparison of different deep-learning methods in the Choroid-DS dataset. ChoroidSeg-ViT achieved mDice, mIoU, and mAcc scores of 98.31, 96.62, and 98.29, respectively, outperforming other methods in the choroid layer segmentation task. Notably, the performance of the model is still improved compared to the novel medical hybrid Transformer method Hi-Former.

**Table 1.** Performance Comparison of Different Deep Learning Methods

Method	mDice	mIoU	mAcc
U-Net	96.73	95.38	97.23
DeepLabV3+	97.6	95.41	97.29
PSPNet	97.81	95.79	97.93
SegFormer	97.2	95.35	97.42
Next-ViT	97.78	95.88	97.85
Hi-Former	97.96	96.24	97.63
ChoroidSeg-ViT	98.31	96.62	98.29

#### Qualitative Results

**Figure 5.**
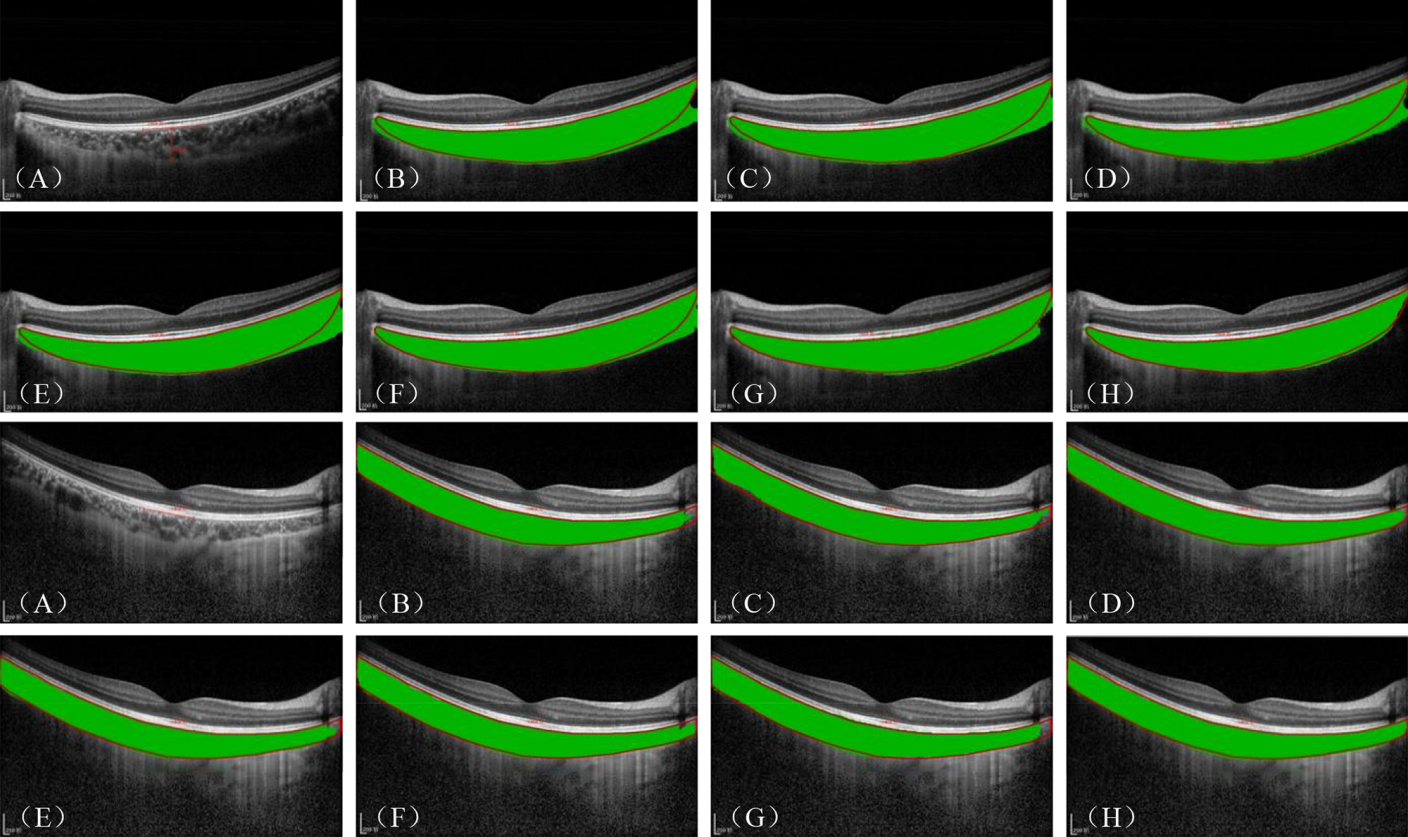
Visualization of choroid layer segmentation results on the Choroid-DS dataset. The results include two examples. (**A**–**H**) Original images and the segmentation performance of U-Net, DeepLabV3+, PSPNet, SegFormer, Next-ViT, Hi-Former, and ChoroidSeg-ViT, respectively. The *green areas* represent the segmentation results, and the *red lines* indicate the ground truth.

Figure 5 presents the visualization results of these comparison experiments. ChoroidSeg-ViT achieved optimal performance in the choroid layer segmentation task. Nevertheless, CNNs such as U-Net and DeepLabV3+ struggle to segment a smooth CSI boundary. This can be attributed to the fact that convolutional models such as U-Net lack the capabilities of multiscale feature extraction and global representation required to accurately define the CSI boundary, leading to under- and oversegmentation issues. In contrast, PSPNet achieves relatively reasonable segmentation results by introducing multiscale fusion features and contextual information. With their excellent global feature extraction capability, the Transformer-based methods emphasize shape features of the choroid layer and segment a smoother choroid boundary. Although SegFormer and Next-ViT segment relatively smooth inferior boundaries, oversegmentation problems occur due to the lack of guidance from local detailed features. In contrast, Hi-Former achieves the closest segmentation performance to ChoroidSeg-ViT by virtue of its ability to express multiscale features. In this study, the proposed model better solved the oversegmentation issues and segmented smoother and more accurate choroid boundaries by introducing the PSCA module, ASFF module, and DFSM, thus enhancing the boundary features as well as the ability to extract multiscale features from different spatial fields and channels. However, ChoroidSeg-ViT suffers from insufficient segmentation detail when dealing with images characterized by high image noise or blurred choroid boundaries, leading to issues such as mis-segmentation in cases where the inferior choroid boundary has low contrast with the background.

### Ablation Study

In this study, Next-ViT was employed as a base model and was improved to achieve better performance. We enhanced the feature extraction ability of Next-ViT as the backbone by modifying the attention module, and introduced a feature fusion path to capture richer semantic information. To understand the impact of each component in ChoroidSeg-ViT on the overall segmentation performance, ablation experiments were conducted on the Choroid-DS dataset. Here, Base represents the base network of the Next-ViT, Ablation-1 indicates the addition of the PSCA module, Ablation-2 denotes the addition of the ASFF module, Ablation-3 represents the combination of ASFF and PSCA modules, and Ablation-4 denotes the integration of all three modules (PSCA, ASFF, and DFSM). Table 2 presents the ablation results obtained from the ChoroidSeg-ViT model on the Choroid-DS dataset.

**Table 2.** Experimental Results of Different Ablation Methods

Method	PSCA	ASFF	DFSM	mDice	mIoU	mAcc
Base	×	×	×	97.78	95.88	97.85
Ablation-1	✓	×	×	98.17 (0.39↑)	96.29 (0.41↑)	97.98 (0.13↑)
Ablation-2	×	✓	×	98.09 (0.31↑)	96.25 (0.37↑)	98.06 (0.21↑)
Ablation-3	✓	✓	×	98.22 (0.44↑)	96.51 (0.63↑)	98.19 (0.34↑)
Ablation-4	✓	✓	✓	98.31 (0.53↑)	96.62 (0.74↑)	98.29 (0.44↑)

Ablation-1 outperformed the base network by 0.39 in mDice and 0.41 in mIoU. This improvement can be attributed to compensating the absence of guidance from multi-scale features in base network. The base network only employs a single group convolution to extract local features, leading to semantic inconsistency between local features extracted by a convolutional layer with a small receptive field and global features captured by the Transformer. Consequently, oversegmentation or missed segmentation issues arise. In contrast, PSCA extracts multiscale features via convolutional blocks of varying sizes and dimensions. This encoding of feature information from multiple dimensions within the region of interest enhances the segmentation accuracy for the choroid boundary.

Also, the mDice, mIoU and mAcc metrics for Ablation-2 exhibited improvements of 0.31, 0.37, and 0.21, respectively, compared to the original network. Such improvement indicates that fusing encoder features from different stages can effectively enhance the segmentation performance of the Transformer architecture. Consequently, the ASFF module is used to enhance the multiscale feature interaction capability of the model by fusing multiscale features from adjacent stages. Simultaneously, integrating features from adjacent layers reduces semantic conflicts caused by significant differences in feature scales, enabling the model to more efficiently utilize the multiscale features extracted by the encoder.

Finally, the base network was improved by adding the ASFF and PSCA modules in Ablation-3, resulting in mDice and mIoU metrics of 98.22 and 96.51, respectively. Further improvement was obtained in Ablation-4 with the help of the DFSM, and optimal segmentation results were obtained for the mDice, mIoU, and mAcc metrics of 98.32, 96.58, and 98.29, respectively. These scores are 0.53, 0.74, and 0.44 higher, respectively, than those achieved by the base network.

**Figure 6.**
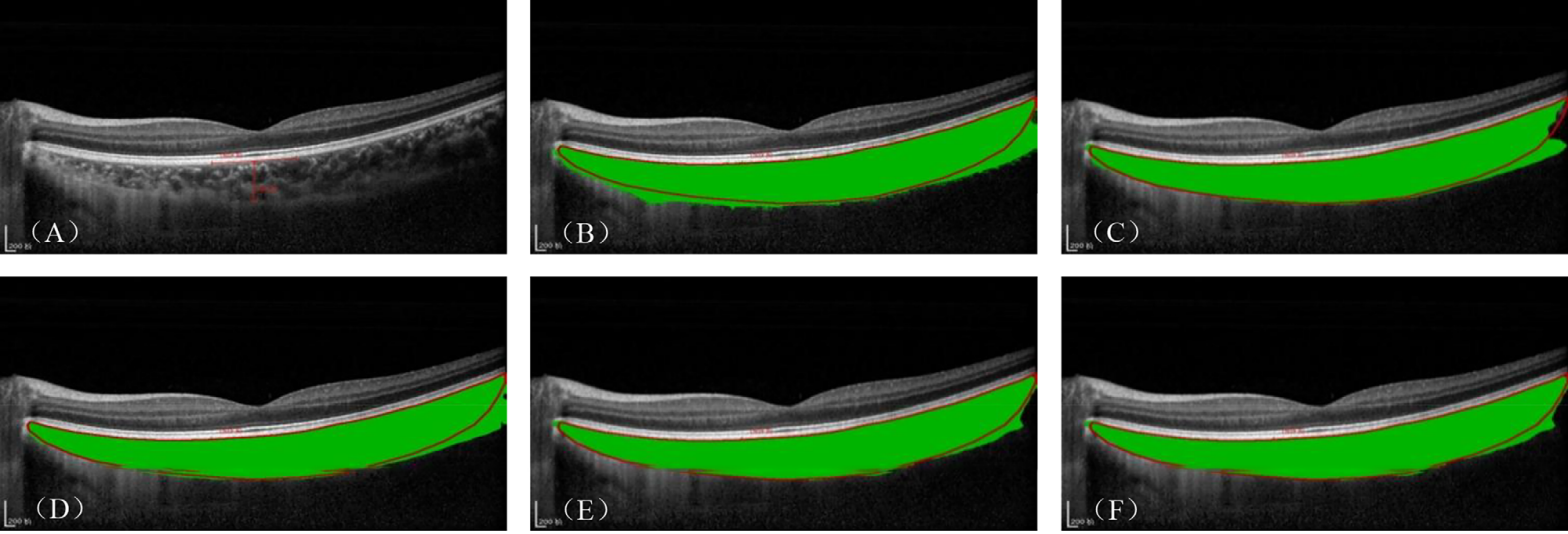
Visualization of the choroid layer segmentation results from ablation experiments. (**A**–**F**) Original images and the segmentation performance of Base, Ablation-1, Ablation-2, Ablation-3, and Ablation-4, respectively.

Visualization results of the ablation studies are shown in Figure 6. Ablation-1 and Ablation-2 incorporate the PSCA and ASFF modules, respectively, which improved the segmentation performance of the choroid inferior boundary compared to the base network. However, they differed from the ground truth due to the issue of oversegmentation. Ablation-3 integrated both the ASFF and PSCA modules, outperforming Ablation-1 and Ablation-2 and resulting in relatively smooth and accurate choroid segmentation boundaries. Based on Ablation-3, Ablation-4 adds the DFSM to improve the semantic consistency of the features, achieving the choroid boundary closest to the ground truth. This superior performance can be attributed to the fact that the PSCA module enhances the multiscale feature extraction capability of the model when combined with the self-attention mechanism to model global and local information. Additionally, the ASFF module, together with the DFSM, fuses encoder output features and dynamically selects multiscale features with same semantic information, minimizing information bias and improving semantic consistency.

Additionally, we compared DFSM with other attention modules to demonstrate the effectiveness of DFSM. Specifically, Ablation-3 was employed as the baseline network, and the squeeze-and-excitation (SE) module, CBAM, and DFSM were incorporated into the Ablation-3 using the same parameter magnitude and training strategy. As shown in Table 3, the proposed DFSM achieved the best performance on the Choroid-DS dataset, outperforming both the SE module^38^ and the CBAM, thus validating the efficacy of our attention module.

**Table 3.** Experimental Results of Different Attention Modules

Method	mDice	mIoU	mAcc
Ablation-3	98.22	96.51	98.19
Ablation-3 + SE	98.15	96.49	98.15
Ablation-3 + CBAM	98.26	96.51	98.24
Ablation-3 + DFSM	98.31	96.62	98.29

Although the SE can assign different attention weights to each feature channel to capture important features in different channel dimensions, it neglects spatial relationships. On the other hand, the CBAM applies both channel and spatial attention, but computing spatial attention to channel-compressed features ignores spatial feature differences between channels. Therefore, considering both channel and spatial information, the proposed DFSM further incorporates pointwise attention, enabling the model to dynamically select information with high semantic consistency. By integrating these three different attention mechanisms, the DFSM enhances the representational capabilities of the model and outperforms both SE and CBAM.

### Generalization Analysis

This study conducted generalization experiments that incorporated the ASFF module, PSCA module, and DFSM into various backbone networks to confirm the reasonableness and generalization of each module. To ensure the validity of the experimental results, generalization analysis was made on other popular CNN and ViT models that are widely used for vision tasks, including U-Net and SegFormer, as well as the benchmark network Next-ViT. As presented in Table 4, the generalization results demonstrate that the modules proposed in this study can be applied to different backbones and significantly improve their segmentation performance for the choroid layer segmentation task.

**Table 4.** Results of Generalization Analysis on Different Backbones

Method	mDice	mIoU	mAcc
U-Net	96.73	95.38	97.23
U-Net + ASFF + PSCA + DFSM	97.59	96.18	98.07
SegFormer-B1	97.2	95.35	97.42
SegFormer-B1 + ASFF + PSCA + DFSM	97.96	96.45	98.05
Next-ViT	97.78	95.88	97.85
ChoroidSeg-ViT	98.31	96.62	98.29

The segmentation performance of U-Net was significantly improved after the proposed modules were added, with improvements of 0.86, 0.8, and 0.84 in the mDice, mIoU, and mAcc metrics, respectively. This improvement can be attributed to the enhanced multiscale representation ability of the U-Net and its ability to extract abstract features in the choroid segmentation task. The proposed modules significantly improved the mDice, mIoU, and mAcc metrics of SegFormer by 0.76, 1.1, and 0.63, respectively. Original SegFormer relies only on FFN to encode the position of image features, making it challenging to accurately locate key features in complex medical images and resulting in relatively low segmentation metrics. The incorporation of the designed modules addresses the issue of lacking sufficient spatial inductive bias, whereas the ASFF module and DFSM enhance its multiscale feature learning and fusion capability.

The study conducted additional cross-dataset generalization experiments on the Retinal OCT-C8 to further evaluate the robustness and generalization capabilities of our model. These experiments aimed to validate the performance of the model on varying resolutions of images depicting diverse lesion types, thus providing insights into its generalization capacity across diverse datasets. According to Table 5, the Choroid-ViT model achieved optimal segmentation performance, reaching 91.78, 95.59, and 95.67 in the three metrics of mIoU, mDice, and mAcc, respectively. This indicates that the present model can be well generalized to the task of segmentation of different diseased choroids at low resolution.

**Table 5.** Performance Comparison of Different Methods on Retinal OCT-C8 Dataset

Method	mDice	mIoU	mAcc
U-Net	94.47	89.86	93.94
DeepLabV3+	94.61	90.08	95.28
PSPNet	94.91	90.59	94.39
SegFormer	95.16	90.89	94.77
Next-ViT	95.17	91.05	95.31
Hi-Former	95.06	90.92	95.25
ChoroidSeg-ViT	95.59	91.78	95.67

**Figure 7.**
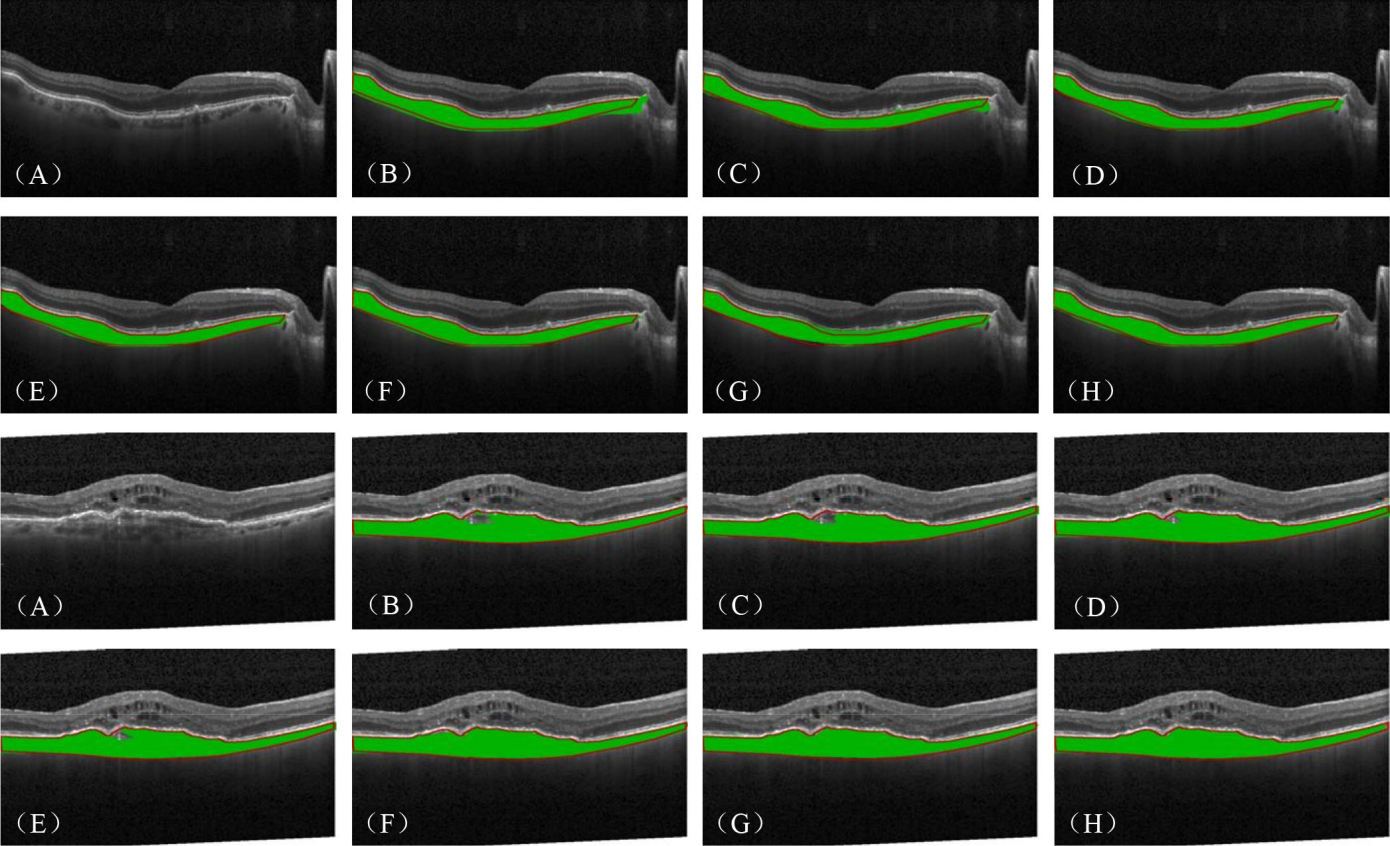
Visualization of choroid layer segmentation results for the Retinal OCT-C8 dataset. The results include two examples. (**A**–**H**) Original images and the segmentation performance of U-Net, DeepLabV3+, PSPNet, SegFormer, Next-ViT, Hi-Former, and ChoroidSeg-ViT, respectively.

In order to demonstrate the segmentation effect of existing methods on the Retinal OCT-C8 dataset in a more intuitive manner, two types of lesion images are visualized in Figure 7: DME and choroidal vitreous wart lesion images. The visualization results demonstrate that the proposed model achieved superior segmentation results, producing segmentation boundaries that are closer to the ground truths while reducing the under- and oversegmentation issues.

## Discussion

This study proposed a model for choroidal layer segmentation in OCT images and analyzed its effectiveness through qualitative and quantitative experiments. The experimental results indicate that ChoroidSeg-ViT is currently the most effective model for this task.

With the emergence and development of deep-learning technology, various segmentation algorithms have been developed and applied to the choroid layer segmentation task. However, the choroid layer in OCT images presents challenges such as fuzzy lower boundaries and poor contrast between the lumen area within the choroid and the background. Traditional CNNs rely solely on convolution algorithms to extract local features, which is not conducive to segmenting large choroidal layers. U-Net improves segmentation accuracy by fusing low-level and high-level features through skip connections. However, direct merging of encoder and decoder features can lead to semantic ambiguities between different scales of the features. DeepLabV3+ and PSPNet introduce multiscale features in different ways to improve their segmentation performance. However, these methods are not suitable for choroidal layer segmentation and result in poor generalization.

Over the past few years, ViTs have become increasingly popular for segmentation tasks due to their exceptional global representation capabilities. ViTs are based on MHSA to achieve comprehensive receptive field coverage for long-term relationship modeling. However, the lack of inductive bias inherent in convolutions limits the generalization ability of the model, which requires a large number of training samples and data enhancement strategies to compensate. Both SegFormer and Next-ViT attempt to enhance the Vision Transformer architecture local feature extraction ability by incorporating convolution modules and guiding global feature learning with the aid of local features. SegFormer achieves this by adding a FFN after the self-attention module to guide the segmentation location, and Next-ViT connects the convolutional and self-attention blocks in series to improve the local and global feature extraction ability. However, these methods lack the capability for multiscale feature extraction and are difficult to apply to the segmentation of the choroid layer with a fuzzy inferior boundary. As a novel and efficient medical Transformer method, Hi-Former highlights the importance of multiscale learning, but its segmentation performance on multi-organ datasets has failed to generalize to the choroid segmentation task.

To address the aforementioned issues, this study proposes ChoroidSeg-ViT. First, the PSCB module is designed to enhance the multiscale learning capability of the model by utilizing convolutional blocks of varying scales and dimensions to extract important features from feature points, space, and channels. Second, the ASFF module is used to fuse the output features of hierarchical encoders. This simple but effective fusion enables the model to fully utilize the multiscale features. Finally, the DFSM dynamically selects fusion features with semantic consistency, which effectively reduces the risk of mis-segmentation. The ablation and comparison experiments support the design of the ASFF module, PSCA module, and DFSM and demonstrate the effectiveness of ChoroidSeg-ViT in choroid layer segmentation. Additionally, the generalization experiments show that the proposed modules have a better generalization ability to various backbones and OCT datasets of different resolutions and pathology images.

The experimental results indicate that ChoroidSeg-ViT achieves superior performance in the choroid layer segmentation task. However, the visualization results reveal an inconsistency problem with the ground truth when segmenting the CSI of the choroid and the lower right boundary. In addition, ViTs have a larger number of parameters compared to CNNs. Although the ASFF module, PSCB module, and DFSM have tried to reduce the computational burden through depth-separable convolution and dropout layers, they inevitably increase the number of parameters. Further investigation is needed to determine how to strike a good trade-off between the number of parameters and segmentation accuracy.

## Conclusions

This study has proposed a local-enhanced Transformer model we refer to as ChoroidSeg-ViT, which is designed for choroid layer segmentation in OCT images. The model is based on a local enhanced feature extraction path that combines the advantages of both Transformers and CNNs, enhancing the capability of the model to extract local and global features. Additionally, the ASFF module and DFSM are designed to serve as the semantic feature fusion path, enabling the gradual fusion of semantic information from features in shallow and deep layers while dynamically selecting significant features with semantic consistency. This approach effectively mitigates semantic ambiguity and eliminates information redundancy. The comparison experiment results demonstrate that ChoroidSeg-ViT achieves superior segmentation performance for the choroid layer segmentation task. Furthermore, ablation and generalization experiments have validated the reasonableness of the module design.
